# Increased risk of venous thromboembolism in patients with granulomatosis with polyangiitis: A population-based study

**DOI:** 10.1371/journal.pone.0270142

**Published:** 2022-06-17

**Authors:** Shelby Marozoff, Alice Mai, Natasha Dehghan, Eric C. Sayre, Hyon K. Choi, J. Antonio Aviña-Zubieta

**Affiliations:** 1 Arthritis Research Canada, Vancouver, British Columbia, Canada; 2 Division of Rheumatology, Department of Medicine, University of British Columbia, Vancouver, British Columbia, Canada; 3 Division of Rheumatology, Allergy, and Immunology, Department of Medicine, Massachusetts General Hospital, Harvard Medical School, Boston, Massachusetts, United States of America; 4 The Mongan Institute, Massachusetts General Hospital, Harvard Medical School, Boston, Massachusetts, United States of America; Nippon Medical School, JAPAN

## Abstract

We assessed the risk and time trends of venous thromboembolism (VTE) including pulmonary embolism (PE) and deep venous thrombosis (DVT) in new granulomatosis with polyangiitis (GPA) cases compared to the general population. Using a population-level database from the entire province of British Columbia, Canada, we conducted a matched cohort study of all patients with incident GPA with up to ten age-, sex-, and entry time-matched individuals randomly selected from the general population. We compared incidence rates of VTE, PE, and DVT between the two groups, and calculated hazard ratios (HR), adjusting for relevant confounders. Among 549 individuals with incident GPA (57.6% female, mean age 55.4 years), the incidence rates for VTE, PE, and DVT were 7.22, 2.73, and 6.32 per 1,000 person-years, respectively; the corresponding rates were 1.36, 0.74, and 0.81 per 1,000 person-years among the 5,490 non-GPA individuals. Compared with the non-GPA cohort, the fully adjusted HRs among GPA patients were 2.90 (95% CI, 1.10–7.64), 4.70 (95% CI, 1.74–12.69), and 1.66 (95% CI, 0.52–5.27) for VTE, PE, and DVT, respectively. The risks of VTE, PE, and DVT were highest during the first year after GPA diagnosis with HR (95% CI) of 11.04 (1.37–88.72), 26.94 (4.56–159.24), and 2.68 (0.23–31.21), respectively. GPA patients are at significantly increased risk of PE, but not DVT. Monitoring for these complications is particularly warranted in this patient population, especially early after diagnosis.

## Introduction

Granulomatosis with Polyangiitis (GPA), formerly known as Wegener’s granulomatosis, is one of the antineutrophil cytoplasmic antibody (ANCA)-associated vasculitides (AAV). Other AAVs include microscopic polyangiitis (MPA), eosinophilic granulomatosis with polyangiitis (EGPA), and renal-limited vasculitis [1]. These severe and multisystemic conditions are characterized by small- to medium-blood vessel inflammation and necrosis involving the respiratory tract, kidneys, and peripheral nervous system, and are associated with the presence of ANCAs directed against proteinase-3 (PR3) or myeloperoxidase (MPO). Although modern treatments have greatly improved survival [2–4], long-term morbidity and mortality are still significant in GPA [5, 6]. Venous thromboembolism (VTE) is a significant contributor to this morbidity and mortality in selected populations of GPA [7–11]. The mechanism of this elevated risk appears to be multifactorial. Systemic inflammation can cause a generalized prothrombotic state by upregulating procoagulants, downregulating anticoagulants, and inhibiting fibrinolysis, and causing widespread endothelial dysfunction [12–17].

A higher incidence of VTE has been reported in systemic lupus erythematous (SLE) [18, 19], rheumatoid arthritis (RA) [20, 21], dermatomyositis (DM) [22], Sjogren’s syndrome [23], and several types of vasculitis [8, 24–26]. A higher incidence of VTE has also been reported in a cohort of GPA patients identified from a British primary care research database [27]. To date, however, we are not aware of any general population data on VTE outcomes among patients with newly diagnosed GPA [7–11, 28–30].

Using a province-wide database of all inpatient and outpatient medical visits from the British Columbia (BC) healthcare system, the aim of our study was two-fold: (1) To estimate the risk of VTE, including PE and DVT, in incident GPA cases, and (2) To assess the risk of PE and DVT in relation to time since GPA diagnosis.

## Materials and methods

### Data sources

Universal health coverage is available for all residents in British Columbia, Canada (population ~ 4.7 million in 2014). Population Data BC captures all provincially funded health care services since 1990, including all outpatient medical visits, [31] hospital admissions and discharges, [32] interventions, [31] investigations, [31] demographic data, [33] cancer registry, [34] and vital statistics [35]. Furthermore, Population Data BC encompasses the comprehensive prescription drug database PharmaNet, [36] which includes dispensed medications for all BC residents since 1996, regardless of source of funding. PharmaNet includes only medications dispensed in community pharmacies, but excludes medications administered to patients when admitted to hospital or to hospital in-patients. Several studies have been successfully conducted based on this database [18, 37, 38].

### Study design

Using Population Data BC, we conducted a matched cohort analysis of incident VTE, PE, and DVT–wherein VTE is a combined outcome of PE or DVT–among individuals with incident GPA (GPA cohort), as compared with age-, sex-, and entry-time-matched individuals without GPA randomly selected from the general population (comparison cohort).

We created an incident GPA cohort with adult cases (>18 years of age) diagnosed for the first time between January 1997 and December 2014. Individuals were identified as GPA if they had two or more physician visits or hospitalizations at least two months apart within a two-year period with an International Classification of Diseases, Ninth Revision, Clinical Modification (ICD-9-CM) or ICD-10-CM code for GPA (ICD-9-CM 446.4 or ICD-10-CM M31.3). To ensure incident GPA patients, we required all incident GPA patients to have at least seven years of prior registration in the databases without a GPA diagnosis.

The validity of ICD-9-CM codes to identify GPA patients in administrative health databases has been demonstrated previously [39, 40]. In these studies, 89% of the patients identified with the diagnosis of GPA through administrative databases were confirmed on chart review to meet American College of Rheumatology criteria for diagnosis.

For the comparison cohort, we matched up to 10 individuals, randomly selected from the general population, without GPA to each GPA patient based on age, sex, and calendar year of study entry (comparison cohort).

Our study cohorts spanned the period of January 1, 1997 to December 31, 2014. Individuals entered the GPA cohort after all inclusion and exclusion criteria had been met. Participants were followed until they either experienced an outcome (VTE, PE or DVT), died, un-enrolled from the health plan through leaving the province (~2% of cases and 5% of controls), or the follow-up period ended (December 31, 2014), whichever occurred first.

### Ascertainment of PE or DVT

The primary outcomes were the first ever PE or DVT event occurring during the follow-up period. Individuals were considered ‘at risk’ and entered into PE analyses if they had not experienced a PE event before, similarly for DVT and VTE. Incident PE and DVT cases were defined by a corresponding ICD code and prescription of anticoagulant therapy (heparin, warfarin sodium, or a similar agent) [41]. We identified PE (ICD-9-CM: 415.1, 673.2, 639.6; ICD-10-CM: O88.2, I26) and DVT cases (ICD-9-CM: 453; ICD-10-CM: I82.4, I82.9) from outpatient and hospitalization data. The anticoagulant therapy prescription was required to be within 30 days prior to and 180 days after the corresponding ICD code for VTE, PE, or DVT. Since VTE is a potentially fatal disease, we also included patients with a fatal outcome. As a patient may have died before anticoagulation treatment, patients with a recorded code of PE or DVT were included in the absence of recorded anticoagulant therapy if there was a fatal outcome within 1 month of diagnosis. These definitions have been successfully used in previous studies and found to have a positive predictive value of 94% in a general practice database [41].

### Assessment of covariates

Covariates consisted of risk factors for VTE assessed during the year before the index date. These included healthcare utilization (outpatient and hospital visits), medication use (COX-2 inhibitors, aspirin, oral contraceptives, hormone replacement therapy, glucocorticoids), and relevant medical conditions using ICD codes (fractures, inflammatory bowel disease, varicose veins, sepsis, hypertension, alcoholism with liver disease), trauma, and surgeries. In addition, the modified Charlson comorbidity index for administrative data was calculated in the year before the index date to further assess the level of pre-existing comorbidity [42, 43].

### Statistical analysis

We compared baseline characteristics between the GPA and comparison cohorts. Continuous variables were compared via two-sample t-tests, while binary or categorical data were compared between groups via chi-square tests of association. We identified incident cases of PE and DVT during the follow-up period and calculated incidence rates (IRs) per 1,000 person-years for these two outcomes individually as well as VTE (i.e., PE or DVT). We estimated the risk-of-death-adjusted cumulative incidence of each event by using the SAS macro CIF and graphically presented these results [44]. The CIF macro implements nonparametric methods for estimating cumulative incidence functions with competing risks data [45, 46]. The macro can also be used to test the hypothesis that cumulative incidence functions are identical across groups. We used Cox proportional hazard regression models to assess the risk of PE and DVT associated with GPA after adjusting for covariates. We entered confounders one at a time into the Cox models in a forward selection according to each confounder’s impact on the hazard ratio (HR) of GPA, relative to the HR in the model selected in the previous step. Cut-off for minimum important relative effect at each step was set at 5%. In order to evaluate the impact of duration of GPA (i.e., follow-up time after GPA diagnosis), we estimated the age and sex adjusted HR and 95% confidence intervals (CI) yearly for the first five years. We used SAS V.9.4 (SAS Institute, Cary, North Carolina, USA) for all analyses. For all HRs, we calculated 95% confidence intervals (CIs). All p values are two-sided.

### Sensitivity analyses

We performed two sensitivity analyses to test the robustness of our results. First, we estimated the cumulative incidence of each event accounting for the competing risk of death according to Lau et al. [47]. Second, we also determined the potential impact that a hypothetical unmeasured confounder might have had on our estimates of the association between GPA and the risk of PE and DVT [48]. We simulated unmeasured confounders with their prevalence ranging from 10% to 30% in the GPA and control cohorts, and odds ratios (ORs) ranging from 1.3 to 3.0 for the associations between the unmeasured confounder and PE/DVT.

### Ethics approval

No personal identifying information was made available as part of this study. Procedures used were in compliance with British Columbia’s Freedom of Information and Privacy Protection Act. Ethics approval was obtained from the University of British Columbia’s Behavioral Research Ethics Board [H15-00887]. This study exclusively used de-identified administrative data, so the need for informed consent was waived by the ethics committee. All data were fully anonymized before the study team accessed them.

## Results

### Baseline characteristics

Our primary analysis included 549 incident GPA cases (mean age 55.4 years; 57.6% female). They were 10:1 matched by age, sex, and study entry date to 5,490 randomly selected controls from the general population. **Table 1** summarizes the baseline characteristics of the GPA cohort compared with the non-GPA cohort. Compared with the non-GPA group, the GPA group had a higher incidence of inflammatory bowel disease, sepsis, hypertension, and trauma, higher Charlson Comorbidity Index, and increased use of COX-2 inhibitors, aspirin, and glucocorticoids. They also had more hospitalizations and healthcare visits in the previous 12 months.

**Table 1:** Characteristics of GPA and comparison cohorts at baseline.

Variable	GPA	Non-GPA	p-value
	N = 549	N = 5490	
Female	316 (57.6)	3160 (57.6)	1
Age, years, mean (SD)	55.4 (15.6)	55.5 (15.6)	0.974
No. outpatient visits, mean (SD)	36.3 (25.7)	8.8 (12.0)	<0.001
Hospitalized	370 (67.4)	865 (15.8)	<0.001
COX-2 inhibitors	24 (4.4)	155 (2.8)	0.047
Aspirin	25 (4.6)	81 (1.5)	<0.001
Oral contraceptives	22 (4.0)	157 (2.9)	0.145
Hormone replacement therapy	25 (4.6)	184 (3.4)	0.142
Glucocorticoids	432 (78.7)	234 (4.3)	<0.001
Charlson Comorbidity Index, mean (SD)	1.66 (2.09)	0.31 (0.96)	<0.001
Surgery	<5	27 (0.5)	0.75
Fractures	11 (2.0)	62 (1.1)	0.096
Trauma	6 (1.1)	12 (0.2)	0.004
Inflammatory bowel disease	9 (1.6)	19 (0.4)	<0.001
Varicose veins	8 (1.5)	52 (1.0)	0.255
Sepsis	41 (7.5)	33 (0.6)	<0.001
Hypertension	164 (29.9)	1229 (22.4)	<0.001
Alcoholism with liver disease	<5	30 (0.6)	0.764

Values are N (percentages) unless otherwise noted.

Abbreviations: COX-2, cyclooxygenase-2; GPA, granulomatosis with polyangiitis; SD, standard deviation.

### Association between GPA and incident VTE

**Fig 1:**
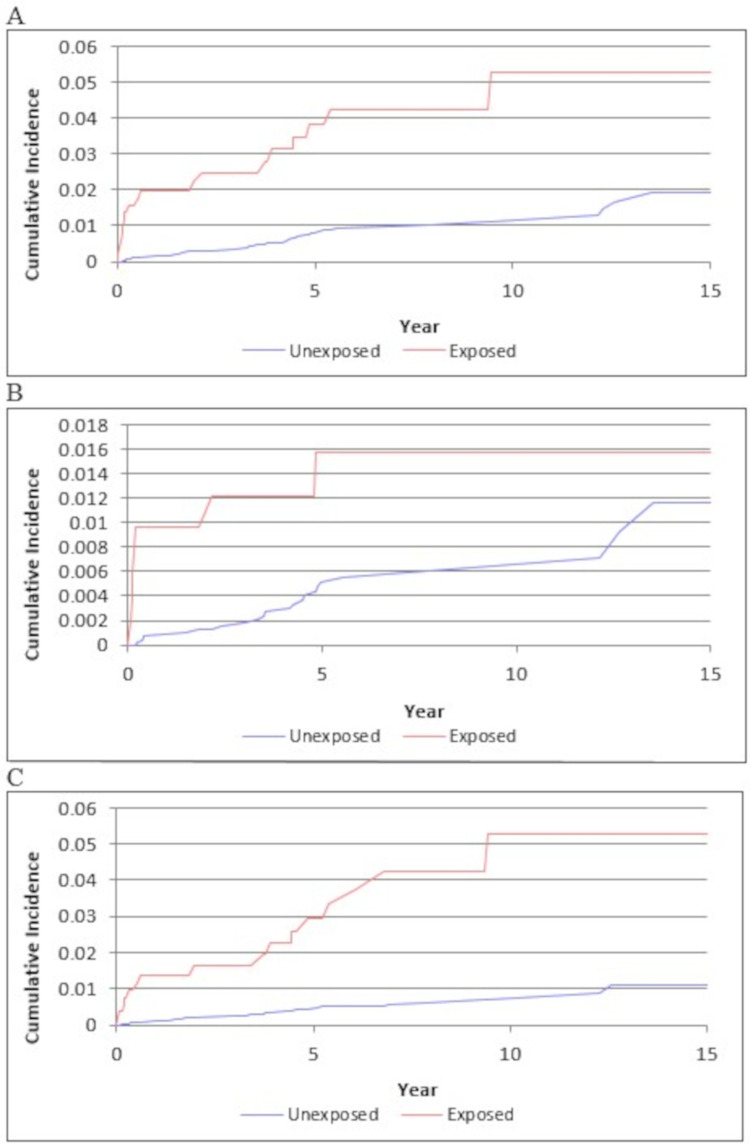
Risk-of-death-adjusted cumulative incidence of venous thromboembolism among granulomatosis with polyangiitis and non-granulomatosis with polyangiitis cohorts. (A) Venous thromboembolism. (B) Pulmonary embolism. (C) Deep vein thrombosis.

GPA was associated with a higher incidence of VTE, PE, and DVT, with incidence rate per 1,000 person-years of 7.22, 2.73, and 6.32, respectively (**Table 2** and **Fig 1**) versus 1.36, 0.74, and 0.81 in the comparison cohort, respectively. The age, sex, and entry time-matched HRs and 95% CIs among GPA patients compared to the comparison cohort were 6.40 (3.55–11.53), 5.67 (2.28–14.09), and 7.97 (4.14–15.35) for VTE, PE, and DVT, respectively. After further adjusting for confounders, the HRs for VTE and PE remained significant at 2.90 (1.10–7.64) and 4.70 (1.74–12.69), but the fully adjusted HR for DVT at 1.66 (0.52–5.27) was no longer significant (**Table 2**).

**Table 2:** Relative risk of incident PE and DVT according to GPA status.

	GPA	Non-GPA
	N = 549	N = 5490
VTE		
At risk	523	5458
Cases, N	18	40
Incidence Rate/1000 Person-Years	7.22	1.36
Age-, sex-, entry time-matched HR (95% CI)	6.40 (3.55–11.53)	1.00
*Fully adjusted age-, sex-, entry time-matched HR (95% CI)	2.90 (1.10–7.64)	1.00
PE		
At risk	529	5475
Cases, N	7	22
Incidence Rate/1000 Person-Years	2.73	0.74
Age-, sex-, entry time-matched HR (95% CI)	5.67 (2.28–14.09)	1.00
*Fully adjusted age-, sex-, entry time-matched HR (95% CI)	4.70 (1.74–12.69)	1.00
DVT		
At risk	531	5468
Cases, N	16	24
Incidence Rate/1000 Person-Years	6.32	0.81
Age-, sex-, entry time-matched HR (95% CI)	7.97 (4.14–15.35)	1.00
*Fully adjusted age-, sex-, entry time-matched HR (95% CI)	1.66 (0.52–5.27)	1.00

*Fully adjusted models include the following selected covariates: for VTE, glucocorticoids, no. outpatient visits, aspirin, hospitalization; PE, hospitalization; DVT, glucocorticoids, no. outpatient visits, aspirin, hospitalization.

Abbreviations: CI, confidence interval; DVT, deep vein thrombosis; GPA, granulomatosis with polyangiitis; HR, hazard ratio; PE, pulmonary embolism; VTE, venous thromboembolism

The time-based risk after diagnosis of GPA revealed a significantly higher risk in the first year after diagnosis of GPA for both VTE and PE, but not DVT (**Table 3**). The risk of these outcomes decreased in the subsequent years.

**Table 3:** Fully adjusted HRs for PE, DVT or both (VTE) in GPA according to follow-up period.

Time after diagnosis	VTE	PE	DVT
	HR (95% CI)	HR (95% CI)	HR (95% CI)
<1 year	11.04 (1.37–88.72)	26.94 (4.56–159.24)	2.68 (0.23–31.21)
<2 years	4.15 (0.81–21.28)	11.37 (2.57–50.32)	1.50 (0.24–9.16)
<3 years	3.74 (0.83–16.94)	8.01 (2.25–28.51)	1.48 (0.24–8.90)
<4 years	3.34 (0.92–12.08)	6.74 (2.08–21.89)	1.62 (0.37–7.19)
<5 years	3.47 (1.20–10.03)	5.09 (1.80–14.38)	2.05 (0.54–7.76)
Total follow-up	2.90 (1.10–7.64)	4.70 (1.74–12.69)	1.66 (0.52–5.27)

Abbreviations: CI, confidence interval; DVT, deep vein thrombosis; GPA, granulomatosis with polyangiitis; HR, Hazard ratio; PE, pulmonary embolism; VTE, venous thromboembolism.

### Sensitivity analyses

HRs from analysis of the association between GPA and PE and DVT that accounted for the competing risk of death remained significant for VTE and PE, and remained non-significant for DVT (**Table 4**). The same was true for the adjusted HRs when assessing for the potential impact of an unmeasured confounder between GPA and VTE, PE, and DVT.

**Table 4:** Sensitivity analyses, HR (95% CI).

Outcome	Primary analysis	Competing risk of death-Cox model	Simulated confounder 10%/OR = 1.3	Simulated confounder 10%/OR = 3.0	Simulated confounder 30%/OR = 1.3	Simulated confounder 30%/OR = 3.0
VTE	2.90	3.08	4.48	3.95	4.47	3.38
	(1.10–7.64)	(1.24–7.65)	(2.30–8.70)	(2.00–7.81)	(2.31–8.68)	(1.72–6.67)
PE	4.70	3.81	4.77	3.52	4.48	3.22
	(1.74–12.69)	(1.43–10.16)	(1.76–12.92)	(1.26–9.86)	(1.65–12.15)	(1.14–9.09)
DVT	1.66	2.02	1.67	1.49	1.65	1.21
	(0.52–5.27)	(0.70–5.85)	(0.53–5.33)	(0.46–4.82)	(0.51–5.29)	(0.36–4.11)

Primary analysis is the fully-adjusted model; Sub-distribution models are adjusted for the same covariates as the primary analysis in addition to age and sex; simulated confounder models included additional covariates per the selection algorithm.

Abbreviations: CI, confidence interval; DVT, deep vein thrombosis; HR, hazard ratio; OR, odds ratio; PE, pulmonary embolism; VTE, venous thromboembolism.

## Discussion

This large population-based study shows that GPA is significantly associated with over a 2-fold increased risk of VTE and over a 4-fold increased risk of PE, as well as a non-statistically significant trend towards an increased risk of DVT in GPA patients. These associations were independent of relevant risk factors available in our database, and provide first evidence at the general population level that PE is an important complication of GPA.

Our findings are largely congruent with those from previous studies. Several retrospective cohort studies of patients with various AAVs also found a high incidence of VTE [7, 8, 11, 49]. A Danish study found that the incidence rate ratios were 25.7 (95% CI, 6.9–96) and 20.2 (95% CI, 5.1–8.1) for PE and DVT, respectively, among a cohort of GPA patients within two years of GPA diagnosis from a rheumatology tertiary care center, when compared with population controls [10]. A British study using a primary care research database found a hazard ratio of 5.24 of VTE in the first 3 years of GPA diagnosis [27]. Borowiec et al. in their analysis of 96 GPA patients identified from a hospital in Poland found that 16.6% of patients experienced a VTE (13 DVT, 2 PE, 1 both DVT and PE) [30]. More recently, in a retrospective analysis of 204 AAV patients identified from a center in West London, including GPA, EGPA, and MPA patients, 14 patients experienced 16 VTE events, with an incidence rate of 1.47 per 100 person-years [28]. This is approximately 20 times greater than the rate of VTE in the general population of the United Kingdom. Notably, VTE incidence was also found to be highest early in disease course; close to one-third of VTE events (31%) occurred within 1 month of AAV diagnosis and 44% occurred in the first year after diagnosis. Also, a similar recent retrospective cohort study of 325 AAV patients in southern Sweden found an incidence rate for VTE of 2.4 per 100 person-years [29]. Neither of the latter two studies provided GPA-specific results.

Similar to previous studies, [7–11, 27, 28] we found the highest incidence of VTE within the first year of GPA diagnosis. This suggests that disease activity or its treatment may be an important contributor to the increased risk. This has been demonstrated for other inflammatory rheumatic and non-rheumatic diseases [18, 50].

The pathogenesis of VTE in GPA is multifactorial and not well understood. Endothelial cell dysfunction is a feature of AAV, likely caused by the interaction between activated neutrophils and endothelial cells [51]. Alterations in procoagulants have also been detected in patients with ANCA-associated vasculitis, but their clinical importance has not yet been elucidated [52]. Classic risk factors such as immobility, family history, and relevant medications and comorbidities were found not to be significantly different among patients with AAV-associated VTE compared to patients without AAV that developed VTE [7]. An increased risk of VTE independent of relevant risk factors has also been seen in other forms of vasculitis [50]. This is consistent with our finding that the risk of VTE remains elevated in GPA patients compared to population controls. Among patients with systemic necrotizing vasculitides, risk factors for the development VTE have been found in previous studies to include older age, male sex, previous VTE, and stroke with motor deficits [8, 9]. Additionally, given that we found a statistically significant increased risk of PE, but not DVT in GPA patients, it is possible that the thrombi associated with a PE event for GPA patients are not generated in the veins of the lower extremities. Instead, they may be formed in the pulmonary arteries. GPA may cause stenosis of the pulmonary artery [53] or thickening of the pulmonary artery wall [54], which may contribute to the risk of PE.

Our study has several limitations. Uncertainty around diagnostic accuracy cannot be completely ruled out; the possibility of misclassification is always present in cases from administrative databases. However, this would be a conservative bias, where ‘false positive cases’ would make the point estimates closer to the null hypothesis. Although a validity study has not been conducted on our identification method, the validity of using ICD codes to identify GPA cases in a Swedish inpatient register has been shown to provide a true positive rate of 88% [55]. Additionally, 70% of GPA cases in our study had at least one ICD code by rheumatologist or in hospital, which indicates increased diagnostic accuracy. GPA cases were also prescribed significantly more rituximab (13.3% of GPA cases versus <0.1% of non-GPA cases, p<0.0001) and cyclophosphamide (38.4% of GPA cases versus 0.2% of non-GPA cases, p<0.0001) following diagnosis. This medication data includes all prescription medications dispensed in community pharmacies, but not medications administered to patients when admitted to hospital or dispensed to hospital in-patients. Our database also lacked some important clinical data, such as body mass index, that may influence the risk for VTE. However, we addressed the potential impact of hypothetical unmeasured confounders in our sensitivity analysis, and the HRs for PE and VTE showed that our estimates were robustly significant. Finally, we did not have access to laboratory data to assess the proportion of MPO-ANCA or PR3-ANCA antibodies in GPA cases and further research is needed to determine if risk of VTE varies according to ANCA type.

Our study has the strength of drawing from data from a population-based sample from both outpatient and inpatient settings. As a result, our findings represent a wider range of GPA patients, and not only the more severe cases as previously studied in cohorts from clinical or hospital-based settings. Thus, our results are generalizable to the general population at large.

## Conclusions

In conclusion, our study provides the first truly population-based evidence of a 190% increased risk of VTE and 370% increased risk of PE in patients with GPA compared with the general population. These findings have important implications for patients with GPA and their treating physicians. These results call for increased vigilance and preventive intervention for patients at high risk, and closer monitoring for VTE in patients with GPA, particularly around the time of active disease. Further studies are needed to investigate the role of anticoagulation in the prevention of VTE in patients with GPA.
